# Oral health assessment in institutionalized elderly: a scoping review

**DOI:** 10.1186/s12903-024-04025-y

**Published:** 2024-02-24

**Authors:** M.H Bakker, M.J de Smit, A. Valentijn, A. Visser

**Affiliations:** 1 Department of Gerodontology, Center for Dentistry and Oral Hygiene, University Medical Center Groningen and University of Groningen, Groningen, The Netherlands; 2 Department of Gerodontology, College of Dental Sciences, Radboud University Nijmegen Medical Center, Nijmegen, The Netherlands

**Keywords:** Oral health, Institutionalized elderly, Patient care

## Abstract

When elderly become frail and in need for complex care, they can no longer live independently at home and may be admitted to nursing homes. Various studies have shown that oral health in this population is remarkably poor, which may lead to distressing situations and impacts quality of life. A variety of definitions or descriptions for oral health is used. Without a uniform parameter, it is impossible to determine whether oral health in institutionalized elderly is actually improving or deteriorating over time, as well as the effect of (preventive) interventions. In search for an adequate and clinically applicable parameter to determine oral health in this specific patient group, this scoping review aims to give an overview of the currently used parameters for determining oral health in institutionalized elderly. Ninety different parameters were identified, and 50 parameters were solely used by one study. Only 4 parameters were frequently used (in > 20 studies). The relevance of these parameters for this specific patient group is discussed. To aid the planning and commissioning of future research and patient care, there is an urgent need for an adequate and uniform parameter for oral health determination in institutionalized elderly.

**Supplementary Information:**

The online version contains supplementary material available at 10.1186/s12903-024-04025-y.

## Background

The elderly population is increasing rapidly. It is estimated that by 2050 the population of the ‘oldest old’ (80 years and over) will be more than tripled [1]. This will have a major impact on healthcare systems, as elderly are susceptible to frailty and care-dependency. Frailty is defined as a state in which elderly are vulnerable to sudden changes in health status because of a decline in physiological function and reserve [2]. Very often, frail elderly have more than one chronic disease (co-morbidity) and show limitations in daily activity (disability) [3].

When elderly become frail and in need of complex care, they can no longer live independently at home and may be admitted to nursing homes. Among these institutionalized elderly oral health is often poor, with high prevalence of caries and radices relictae, accompanied by poor oral hygiene [4–6], oral dryness, oral pain and poor oral function [7]. This can be a major risk factor for general health and quality of life [8, 9]. Poor oral health, especially periodontal disease, is associated with several systemic chronic conditions, such as cardiovascular disease, type 2 diabetes mellitus, rheumatoid arthritis, inflammatory bowel disease, Alzheimer disease, nonalcoholic fatty liver disease, certain cancers and aspiration pneumonia [10, 11]. Loss of teeth or broken teeth can cause chewing problems leading to changes in nutritional intake which can result in an easy to chew diet with low protein and low levels of vitamins [12] and malnutrition [13]. Therefore, in frail elderly, maintaining good oral health is essential for systemic health and quality of life.

An important risk factor for poor oral health is the use of specific medication or combinations of medication, which is frequently seen among older adults. In case of polypharmacy (i.e. 4 or more different medication), the risk of developing a dry mouth is high [14]. A low level or poor quality of saliva rapidly increases the risk for caries and periodontitis [15, 16]. Other risk factors that can contribute to the deterioration of oral health is the reported change in oral care behavior including oral self-care. Among a group of home-dwelling, frail elderly almost half (44%) of the participants reported difficulties with visiting the dentist [17]. There are various reasons mentioned for the decline in dental office visits: low energy, the perceived effort does not weigh up against the perceived efforts, dental fear and the (perceived) lack of availability of dental care [17, 18]. Among institutionalized elderly visiting the dentist is even more problematic, as they fully depend on caretakers to organize oral (self) care [4].

The older institutionalized patient can therefore be considered as particularly vulnerable with a high risk of developing poor oral health in a short period of time. As the elderly population is growing rapidly and people tend to retain their natural teeth until high age, oral health problems in institutionalized elderly will rapidly increase as well. In the past years, there has been a remarkable increase in research articles focusing on oral health in institutionalized elderly, most of them on the prevalence of oral health problems. All studies in this domain show comparable results, i.e. poor oral health and poor oral hygiene are omnipresent in institutionalized elderly. Research designed as clinical trials to determine the outcomes of preventive oral health care measures or interventions for institutionalized elderly are rare. Furthermore, studies assessing oral health in institutionalized elderly use a variety of definitions or descriptions for oral health. Although there is consensus on a definition of oral health [19], lack of a universal parameter, or combination of parameters, to assess oral health in institutionalized elderly makes it impossible to compare outcomes of different studies. Even more important: without a clear parameter, it is impossible to determine whether oral health in institutionalized elderly is actually improving or deteriorating over time, as well as the effect of (preventive) interventions. Given the concerns about the effect of poor oral health on quality of life and healthy ageing in a physical and mental context and the newly formulated goals of global institutions as The World Health Organization and The United Nations Decade of Healthy Ageing (2021–2030) [20], this is problematic. In search for an adequate and clinically applicable parameter to determine oral health in institutionalized elderly, this scoping review aims to give an overview of the currently used parameters in literature for determining oral health in institutionalized elderly.

## Methods

This scoping review was executed according to the PRISMA-ScR checklist [21].

The databases MEDLINE, Cinahl and Cochrane Library were searched between January 1st and January 18th 2024 for research articles that reported on oral health in institutionalized elderly. A combination of MeSH Terms and free text words were used:

1. Oral health
2. Elderly, aged
3. Nursing home, institutionalized

As the aim of this study was to provide an overview of all relevant oral health research in institutionalized elderly, and there was merely no research available before 1980’s on this topic, it was chosen to set the time-frame from 1970-on.The online search strategy can be found in the Supplementary file 1.

Selection criteria were: original articles on oral health in institutionalized elderly of which full text was available in English or Dutch. If no full text was available, corresponding authors were contacted by email once. When authors did not respond within 2 weeks, the article was excluded. Reviews were scanned by hand for relevant studies.

Exclusion criteria were: no original articles (i.e. reviews or validation studies), articles reporting on oral health in community-dwelling elderly only, articles of which no full text (in English or Dutch) was available and articles in which oral health was not clearly described. Except for reviews and validation studies, there was no exclusion based on study design. As the main purpose of this study was to determine which parameters for oral health in institutionalized elderly are used in current research, the articles themselves did not undergo a quality assessment. Therefore, no inter-rater reliability was calculated.

After the first screening for relevancy of the abstracts, studies were selected based on the in- and exclusion criteria. Screening of the abstracts and selection of the articles based on the in- and exclusion criteria was done independently by three researchers (AVa, MdS and MB). Afterwards, the results were compared and differences were discussed. Consensus among all three researches had to be reached for an article to be in- or excluded. When no consensus was reached, a fourth researcher (AVi) was consulted.

### Data extraction

All study populations consisted of institutionalized elderly, as this was an inclusion criterion. Oral health assessment was the main variable for which data were extracted. Data-extraction was done in triplicate. Name of the assessment (e.g. DMFT, CPITN etc.), detailed description of the assessment and the assessor were listed. The oral health parameters identified were categorized in 3 categories (objective, subjective or combined parameters) and clustered in subdivisions to facilitate interpretation.

## Results

### Study selection

**Fig.1:**
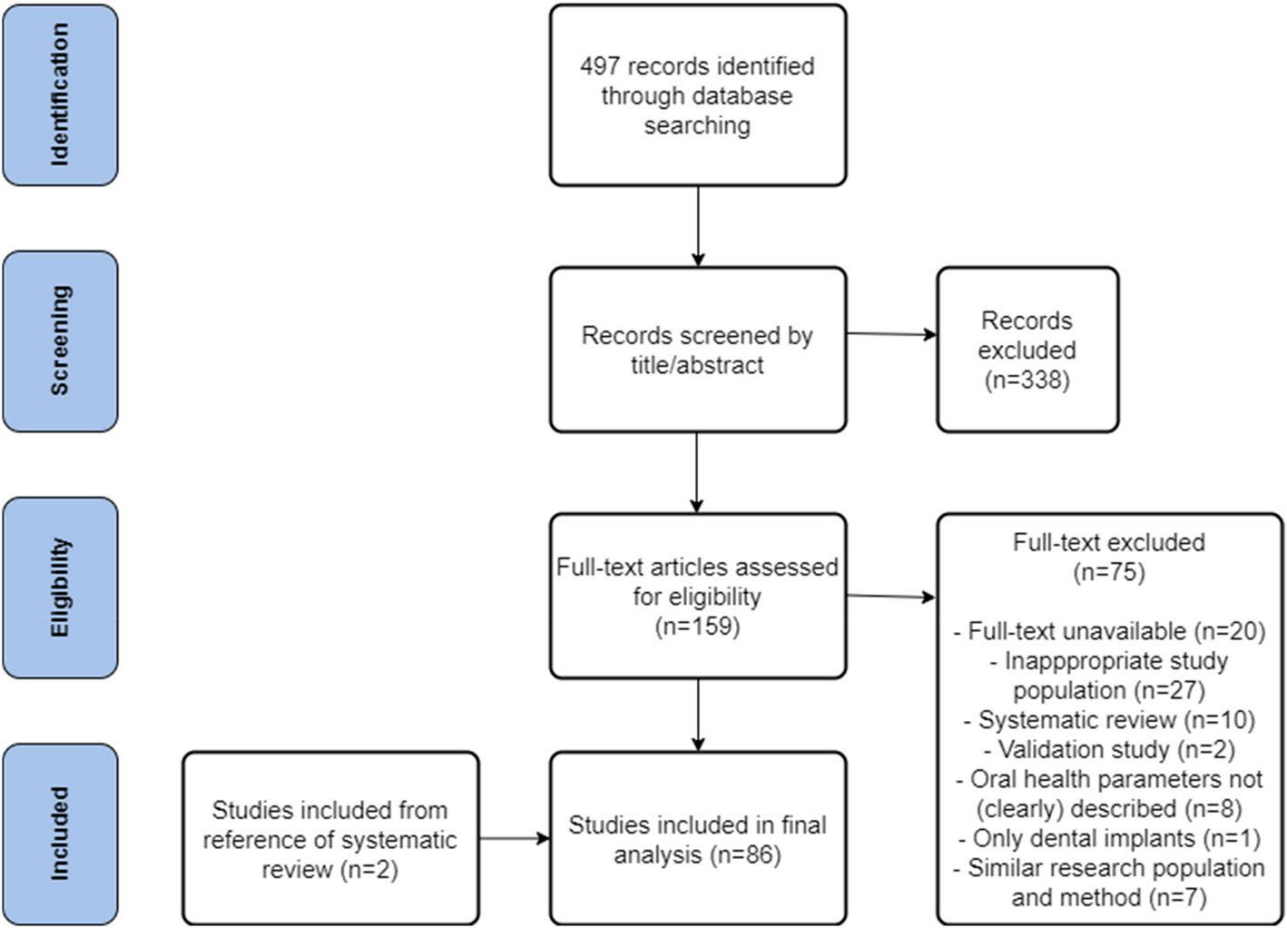
Flow diagram of the selection process

The selection process is shown in Fig. 1. A total of 497 articles were identified (Medline *n* = 343, Cinahl *n* = 5 and Cochrane Library *n* = 149). Duplicates (*n* = 30) were removed. After screening titles and abstracts for relevancy, another 308 articles were excluded. Of the remaining studies, full text was assessed. Full text was unavailable for 20 titles. Twenty-seven studies were excluded as they did not include institutionalized elderly. Systematic reviews (*n* = 10) and validation studies (*n* = 2) were also excluded, however, two studies [22, 23] retrieved from the reference list of the systematic review of Rejnefelt et al. [24] were added. Eight studies were excluded because oral health parameters were not or not clearly described in the methods-section, and one study was excluded because it only focused on dental implants. This resulted in 91 included studies. It appeared that some of the included studies derived from the same study group (same authors) and used the same study population and study protocol (i.e. the same parameters were used to assess oral health). In order to prevent bias to our results, it was decided to cluster these studies into one study per study group. This resulted in 12 studies being clustered into 5 studies (i.e. exclusion of 7 studies) [25–36]. Altogether, 86 studies were included for analysis.

In the decades 1970–2010, 23 studies were included, from 2010 up to 2023, 63 studies were included, indicating increased interest in this topic. Most studies (*n* = 77) were conducted in high-income countries based on the New World Bank country classification (2022–2023) [37], no studies were conducted in low-income countries (for details see Supplementary files 2 and 3).

### Oral health parameters

The definition or description of oral health, the parameter(s) used and the number of studies in which the parameter was used are listed and described in Tables 1, 2 and 3. The following categories were defined:

**Table 1:** Objective parameters

Assessment	Number of unique studies	Studies	Description of assessment
***Dental status***	***63***		
DMFT – decayed, missed, filled teeth	38	[29, 35, 38–73]	Decayed Missed Filled Teeth Index - Based on the presence of teeth and use of dentures, elders were classified as CD: complete dentures, edentulous without CD, partially dentate with prosthesis and partially dentate without prosthesis [38] - Including root caries [52]
DMF(R)S – decayed, missed, filled (root) surfaces	3	[60, 74, 75]	Decayed filled (root) surfaces
Dental status (presence and number of teeth)	21	[5, 27, 40, 45, 57, 71, 76–90]	Presence (0 or 1) of own teeth and/or the number of teeth
Number of occluding pairs	6	[27, 40, 69, 77, 79, 91]	Number of functional occluding pairs with static contacts
Dental treatment need	6	[27, 29, 46, 56, 84, 92]	- Grades 0: no treatment needed, 1: treatment needed [27] - Restorative/prosthodontic/extractions/urgent care [56] - Need for treatment: filling/extraction/denture/other [29, 92] - Treatment need: presence of retained roots, decayed teeth, suspicious changes mucosa or swelling [46] - Dental treatment need: preventive, routine, non-urgent, urgent or immediate emergency [72] - Simple/complex treatment, dental treatment, extractions [84]
Dental risk assessment	1	[63]	Individual dental risk assessment was graded from 1 to 4 according to: general risk (general health, compliance), technical risk (previous dental work), dental caries risk and/or periodontitis risk
Root caries index	5	[39, 50, 74, 86, 93]	Grades 1–5 on buccal side of teeth
Root and/or coronal caries	12	[35, 51, 56, 72, 75, 76, 83–85, 90, 94, 95]	Number of teeth with root caries and/or coronal caries
Clinical dental functionality score	1	[96]	Score based on the number of occluding contacts and whether they are evenly distributed between jaws
***Oral health status***	***4***		
Oral health status, oral care status, oral status	3	[87, 92, 97]	- Oral health status scored as poor/medium/good, based on several clinical aspects (dental visits/oral mucosa condition/presence of teeth) [97] - Oral care status of teeth, mucous membranes and dentures scored as good/fair/poor [92] - Presence of oral status problems: gingivitis, caries, tooth fracture [87]
Oral health index	1	[71]	Oral health index was created: ranging from 0–9, the sum of all parameters. OHI score of less than 3 was acceptable, higher score than 6 was high need for oral care. Parameters: caries/root remnants, periodontium, oral hygiene and denture
***Periodontal parameters***	***42***		
Periodontal status CPITN or CPI	15	[32, 39, 46, 48, 51, 56, 61, 62, 64, 65, 67, 74, 85, 86, 98]	Community Periodontal Index of Treatment Needs: a screening tool to assess presence or absence of periodontal pockets, calculus and gingival bleeding Community Periodontal Index (CPI) is the modified version of CPITN
Periodontal parameters according to National Institute of Dental Research Criteria	1	[53]	Presence of dental plaque, bleeding, calculus, gingival recession, pocketing, level of attachment
Periodontal screening (and recording) index	3	[44, 58, 59]	Score 0–4 for each sextant based on measuring periodontal pockets and the extend of the resulting bleeding
Measuring pocket depth	1	[55]	Measured mesially and distally of all elements, scores clustered in < 4 mm, = 4 mm and > 4 mm
Assessment of periodontal status	1	[45]	Periodontal status described by presence of calculus and bleeding on probing
Dutch Periodontal Screening Index	1	[71]	Each sextant is scored based on pocket depth (range 0–4). Highest score is the patient’s DPSI score
Extent and severity index score	1	[52]	Periodontal score based on the extent (< 30% is localized, > 30% is generalized) and severity (clinical attachment level slightly (1–2 mm), moderately (3–4 mm) or severely (5 mm)
Periodontal disease / tooth mobility	2	[45, 76]	Miller’s classification on tooth mobility
Tooth mobility	1	[55]	Tooth mobility graded in 1: horizontal mobility less than 1 mm, 2: between 1 and 2 mm mobility, 3: horizontal mobility > 2 mm
Calculus index	5	[39, 43, 45, 86, 94]	- Volpe-Manhold Index [39, 86] - Calculus index; ranging 0–3 [43] - Presence/absence calculus [45] - Average calculus score [94]
Gingivitis/periodontitis assessment	1	[95]	Pocket depth > 5.5 mm, bleeding, suppuration and / or tooth mobility class III
Plaque index	21	[5, 32, 39, 41, 50, 55, 58–60, 71, 72, 80–82, 86, 88, 93, 94, 99–101]	- Plaque index grades 0–3 [39, 41, 50, 60, 71, 80, 86, 88, 94, 99, 101] - Plaque index grades 0–2 [5] - Modified plaque index [82] - Quigly-Hein index grades 0–5 [58, 86] - Mucosal plaque index (MPS) – dentate + edentulous, grades 1–4 [93, 100] - Plaque control record or full mouth plaque score (using plaque indicator, calculated percentage) [32, 41, 59] - Approximal plaque index determined in percentages [55]
Bleeding index	7	[32, 35, 45, 55, 86, 93, 98]	- Modified sulcus bleeding index, grades 0–3 [93] - Papilla bleeding index [86] - Gingival bleeding index [32, 35, 98] - Presence of bleeding after probing [45] - Sulcus bleeding index [55]
Gingival/gingivitis index	12	[39, 50, 60, 62, 72, 73, 80, 82, 86, 88, 99, 102]	- Visual appearance of inflammation grades 0–2/0–3 [39, 50, 60, 62, 72, 73, 80, 86, 88, 99, 102] - Modified gingival index [73, 82]
***Oral hygiene***	***23***		
Oral Hygiene Index (OHI)	10	[40, 43, 46, 47, 49, 54, 73, 83, 86, 102]	- OHI: combination of debris index and calculus index for 12 tooth surfaces—grades 0–3 [49, 86] - s-OHI: uses only 6 tooth surfaces [40, 43, 54, 73, 83, 102] - m-OHI: summation of average debris index and calculus index [46] - UM-OHI: using disclosing agent, determines plaque in 12 regions [47]
Denture Hygiene Index	8	[32, 40, 41, 55, 58, 81, 83, 98]	- Grades excellent, fair, poor [40, 83] - Percentage 0–100% [32, 41, 81, 98] - Scoring dentate/mucosal surface of the denture, maximum score 10 [55]
Denture cleanliness	1	[90]	Denture cleanliness was defined as good, medium, poor
Biofilm index for dentures	1	[101]	Score 0–4 for presence of biofilm on the denture in 5 areas
Food debris / debris index	5	[40, 43, 83, 86, 90]	- Food debris after rinsing, 6-point scale [86] - Debris index, ranging 0–3 [40, 43, 83, 90]
Tongue coating index	2	[76, 99]	- Tongue coating coverage, grades 0–4 [99] - Using the classification by Miyazaki [76]
Oral hygiene assessment based on dependency	1	[92]	The extent to which the patient can independently practice oral hygiene
Independence of oral care	1	[76]	The ability to independently perform oral selfcare
Oral hygiene status	2	[35, 87]	- Presence of calculus, plaque and gingival bleeding used for a subjective assessment based on the dentists’ judgement to evaluate oral hygiene status [35] - Oral hygiene status scored based on the presence and amount of calculus [87]
***Denture related parameters***	***35***		
Presence of dentures	23	[27, 29, 38, 40, 42, 45, 48, 51, 60, 64, 65, 67, 70, 73, 77, 78, 81, 85–87, 90, 91, 103]	Presence or absence of removable denture
Denture fit or condition	10	[5, 51, 52, 56, 71, 84, 86, 94, 97, 104]	Fit of the removable denture
Presence and retention/stability	2	[75, 79]	Presence and retention / stability of removable denture
Type, fit and condition of denture	1	[49]	Type, fit and condition of the removable denture by the classification of Vigild
Denture quality	1	[85]	Quality of the removable denture was scored on a gravity scale
Prosthetic need	1	[72]	Prosthetic need was defined as: no prosthesis needed (0), full denture (1) or partial denture needed (2), denture realignment (3)
***Oral function***	***9***		
Masticatory performance / chewing efficiency	3	[27, 38, 59]	Twenty chewing cycles with two-color chewing gum. After flattening, the gum was scanned and colorimetric assessment was performed [38, 59] or a score was given, grades 1–5 [27]
Clinical dental functionality (CDF) score	1	[96]	CDF score is based on the even distribution of functional contacts in the upper and lower jaw
Swallowing threshold	1	[38]	The number of chewing cycles performed by the patient to chew a portion of unsalted roasted peanuts
Swallowing test	1	[76]	Water swallowing test with 3 mL cold water, than swallow twice, grades 1–5
Oral dryness	1	[93]	Mirror-sliding friction test
Dry mouth (wetness tester)	1	[76]	Measuring dry mouth by a new wetness tester, grades 0–3
Salivary secretion/salivary IgA, pH/halitosis and mouth opening	1	[105]	All parameters were measured according to guidelines
Salivary gland flow rates	1	[53]	Unstimulated and stimulated salivary flow was collected using a modified Carlson-Crittenden cup
Krogh-Poulsen test	1	[46]	Test using a flat, thin wedge to determine cracked teeth, damaged dentures, occlusive surfaced and joint pain
***Oral pathology***	***16***		
Stomatitis, presence of denture-related stomatitis	3	[48, 83, 102]	Denture stomatitis grading I – III [48] Presence of denture stomatitis [83, 102]
Prevalence of oral lesions	1	[87]	Presence or mixture of the following lesions: Candidiasis, aphthous ulcer, cheilitis, fistula, abscess, red or white lesion, dry mouth
Presence of oral lesions	1	[49]	Presence or absence of oral pathology, such as swellings, sinus tracts, ulceration and stomatitis
Mucosal lesions	1	[85]	Presence of mucosal lesions: generalized stomatitis, denture-induces ulcers or various
Presence of oral pathology	1	[50]	Presence of denture stomatitis, angular cheilitis, oral ulceration, fissured tongue, red or white lesions
Presence of mucosal pathology	1	[51]	Presence of ulceration, leukoplakia, angular cheilitis, fibrous lesions, denture stomatitis
Oral mucosal lesions	1	[56]	Presence of oral mucosal lesions, tooth defects, bone disorder
Oral soft tissue	1	[90]	The oral soft tissues were examined for the presence of erythema, mucosal plaques, atrophic glossitis, pseudomembranous candidosis, stomatitis, gingivitis, denture induced hyperplasia and denture-induced ulceration
Soft tissue lesions	1	[104]	Presence of soft tissue lesions
Mucosal rating scale	1	[53]	Presence of erythemic or leukoplakic lesions, ulcerations and erosions
Oral mucosa condition	1	[97]	Presence of denture-induced stomatitis, inflammatory papillary hyperplasia, chronic atrophic candidiasis
Alterations of oral mucosa	1	[55]	Alterations of oral mucosa (not further specified)
Treatment need oral mucosa or gingiva	1	[92]	Assessment of care status of mucous membrane (good, medium, poor)
Oral tissue anomalies scale	1	[94]	Based on Roed Peterson and Renstrup [106]; an examiner rates the presence of tissue anomalies. The number of anomalies was summed to create oral tissue anomalies score
***Halitosis***	**2**		
Oral odour Halitosis	2	[107] [76]	Oral odour was examined by opening the mouth and make an ‘ah’ sound for 5 s, grades 0–4 Halitosis was categorized by 6 stages, scores from 3 to 5 indicated the presence of halitosis

**Table 2:** Subjective parameters

Assessment	Number of unique studies	Studies	Description of assessment
**Dry mouth**	**5**		
(Summated) Xerostomia Inventory ((S)XI)	2	[40, 79]	Eleven or 5 items are scored by the patient, grades 1–3 (my mouth feels dry, difficulty eating dry foods, difficulty swallowing foods, lips feel dry)
Xerostomic VAS	1	[86]	Visual Analogue Scale to quantify dry mouth, ranges 0–10
Dry mouth symptoms and oral motor function	1	[105]	Likert scales to rate dry mouth and motor function
Dry mouth scale	1	[94]	Four questions on dry mouth, answered yes or no
***Oral health***	**13**		
Oral symptoms	1	[78]	Chewing and swallowing problems, dry mouth
Assessment of oral health	1	[108]	Standardized questionnaire on problems with eating, chewing and xerostomia
Oral function scale and oral problems self-report and oral hygiene	1	[94]	Degree of satisfaction with oral functioning, rated 1–5, and questionnaires regarding oral problems and oral hygiene
Oral symptoms questionnaire	1	[57]	Oral symptoms: sensitive teeth, toothache, broken teeth, missing teeth, bleeding gums, dry mouth, burning mouth, dry lips
Oral conditions	1	[88]	Questions concerning: pain in the mouth, bleeding gums, tooth mobility, bad breath, burning mucosa, excess saliva, or dryness, swallowing difficulties, pain in the temporomandibular joint
Oral health	2	[64, 67]	Questionnaire on subjective oral health conditions: teeth problems, gum problems, opinion on oral status
OHIP and/or GOHAI	7	[61, 64, 65, 67, 72, 100, 101]	OHIP: Oral Health Impact Profile: a 14-items questionnaire to measure self-reported functional limitation, discomfort and disability to oral conditions GOHAI: Geriatric Oral Health Assessment Index: a 12-items questionnaire to evaluate self-perceived oral health
Dental visit checklist	1	[97]	Checklist including number of dental visits in the last 2 years and reason of last dental visit
***Oral pain***	**5**		
Presence of oral pain in the last 4 weeks	1	[49]	Presence of oral pain
Orofacial-pain scale for non-verbal individuals	1	[40]	Orofacial-pain scale for non-verbal individuals
Tooth/ jaw pain	1	[109]	Presence of tooth or jaw pain
Oral pain	1	[46]	Oral pain experienced in past year
Dental complaints	1	[84]	Presence of dental complaints
***Masticatory function***	**1**		
Masticatory difficulties (VAS 0–10)	1	[59]	Visual Analog Scale to measure masticatory difficulties

**Table 3:** Combined parameters

Assessment name	Number of unique studies	Studies	Description of assessment	Validation
OHAT	8	[27, 41, 103, 107, 110–113]	Oral Health Assessment Tool Eight categories (lips, tongue, gums and tissues, saliva, natural teeth, dentures, oral cleanliness and dental pain) scored as 0 – healthy, 1 – changes or 2 – unhealthy	Yes [114]
ROAG(J)	5	[25, 80, 81, 115, 116]	Revised Oral Assessment Guide – Jonkoping Evaluation oral health by assessing the condition of voice, lips, oral mucosa, tongue, gums teeth, saliva, swallowing, protheses/implants (grades 0–3)	ROAG: yes [117]
ADS	1	[118]	Asymptotic Dental Score – sum of oral pathologies: dental caries or one edentulous jaw (grades 0–3), gingivitis (grades 0–1), root remnants (grades 0–2), number of teeth with pockets (grades 0–3). Low ADS 0–2, moderate ADS 3–4 and high ADS 5–9	No
Oral health examination instrument	1	[109]	An instrument based on OHAT and Oral Health Module. Questions concerning lip health, breath odour, saliva appearance, natural teeth count, gingival inflammation, toot hand jaw pain, presence of dentures, denture fit and hygiene, mucosal status and oral health abnormalities	No
RAI MDS	3	[96, 119, 120]	Resident Assessment Instrument – Minimum Data Set. Oral health problems concerning chewing, swallowing, pain, debris, dentures, teeth lost, broken teeth, inflamed gums, daily oral health care	Inconclusive [121]
BOHSE	1	[122]	Brief Oral Health Status Examination: 10 items reflecting the status of oral health (lips, tongue, tissue inside cheek, floor, roof of the mouth, gums between teeth, saliva, condition of natural teeth, condition of artificial teeth, occluding pairs, oral cleanliness), rated on 3-point scale	Yes [123]

1. Objective parameters (Table 1).

Objective parameters objectively qualify oral health (i.e. without the patient's opinion). These parameters usually focused on one single aspect of oral health. Objective parameters were subdivided into 8 categories to facilitate further interpretation; 1. Dental status, 2. Oral health status, 3. Periodontal parameters including plaque indices, bleeding indices and presence of calculus, 4. Oral hygiene, 5. Denture related parameters, 6. Oral function, 7. Oral pathology and 8. Halitosis.

2) Subjective parameters (Table 2).

Subjective parameters are based on subjective measurements, e.g., oral pain or subjective chewing ability. Subjective parameters were measured by questionnaires or scales, which were completed by the patient or caretaker and varied in length. Subjective parameters were subdivided into the following 4 categories: 1. Dry mouth, 2. Oral health, 3. Oral pain and 4. Masticatory function.

3) Combined parameters (Table 3).

Combined parameters used a variety of objective as well as subjective parameters combined in one instrument to describe oral health. This category included validated (OHAT, ROAG, BOHSE [114, 117, 123]) as well as unvalidated instruments (ADS, Oral health examination status, RAI MDS [121]).

All parameters identified are below described in detail:

1. Objective parameters (Table 1).

### Dental status

In total, 45 unique studies reported on dental status. Dental status was often recorded by the decayed, missed, filled teeth/surfaces (DMFT/S) index. The DMFT/S was used in 38 studies [29, 35, 38–73]. This index reflects oral status by describing how many teeth are decayed, missing and/or filled. In some studies the DMFT was used to classify oral status: natural dentition without dentures or (partial) edentulous with or without dentures [38]. Also root caries can be added to the index [52]. The DF(R)S index (Decayed, Filled, (Root) Surfaces index, can be considered as an alternative to the DMFT/S index, without ‘missing’ surfaces and including root surfaces [60, 74, 75]. These measures require a dental professional for assessment. Another measure used for dental status was the presence and number of teeth. This measure was used in 21 studies [5, 27, 40, 45, 57, 71, 76–90]. In some studies it was combined with counting the number of functional occluding pairs that had static contacts [27, 40, 69, 77, 79, 91]. Some studies assessed dental status by the presence of decay only, by either reporting a root caries index [39, 50, 74, 86, 93] or counting the number of teeth with root and/or coronal caries [35, 51, 56, 72, 75, 76, 83–85, 90, 94, 95]. Another method for assessment of dental status was dental treatment need [27, 29, 46, 56, 84, 92]. The method differed for every study: it could be simply grading 0 (no treatment needed) or 1 (treatment needed) [27] specified which type of treatment was required [29, 46, 56, 92] and if this was simple or complex treatment [84]. One study used the dental risk assessment: a method to assess individual dental risk based on general and technical risk factors and dental caries and periodontitis risk [63].

### Oral health status

Four studies assessed oral health status [71, 87, 92, 97] by four different methods. In one study, oral health status was assessed by a combination of clinical aspects (scored by a dental professional) and the use of a dental visit checklist [97]. There were 3 categories; good, medium or poor, based on the clinical presentation. For instance, edentulous patients without dentures were categorized as poor, partially edentulous patients with 20 occluding contacts were categorized as good. One study assessed oral care status: the dentist determined whether oral care status was good, fair or poor [92]. This was not further specified. The third study assessed presence of oral health status problems: the presence of gingivitis, caries or tooth fracture [87]. The fourth study created an oral health index: a score between 0 and 9 was given, based on the following parameters: caries or root remnants, periodontium, oral hygiene and denture [71].

### Periodontal parameters

Within the great variety of parameters used to define oral health, periodontal parameters were used in 42 unique studies. They are further subdivided in periodontal screening instruments, plaque or calculus indices and bleeding or gingival indices.

### Periodontal screening instruments

The Community Periodontal Index of Treatment Needs (CPITN) was used in 15 studies [32, 39, 46, 48, 51, 56, 61, 62, 64, 65, 67, 74, 85, 86, 98]. The CPITN was designed as a screening instrument enabling the dental professional to get a quick overview of the periodontal status [124]. The CPITN divides the dentition into sextants and provides these sextants with a periodontal health score.

Eleven studies used other periodontal measures: measurements according to the National Institute of Dental Research Criteria [53], the Periodontal Screening Index [44, 58, 59], Dutch Periodontal Screening Index [71], Extent and Severity Index [52] and The Miller Index score [45, 76];

- National Institute of Dental Research Criteria; examination of 6 teeth on 6 sides and assessing presence or absence of dental plaque, gingival bleeding, supra- and subgingival calculus, as well as probing pocket depth [53].
- Periodontal Screening Index [26]: screening 6 points per tooth and per sextant ranging from 0–4 based on probing depth, bleeding on probing and calculus.
- Extent and Severity Index [52]: periodontal score based on the extent of periodontitis (clinical attachment loss categorized slight (1–2 mm), moderate (3–4 mm) or severe (5 mm).
- Dutch Periodontal Screening index [71]: each sextant is scored based on pocket depth (score ranges 0 to 4). The highest score per sextant is recorded.
- The Miller index score; assessment of tooth mobility (grade 0–3). This measure is used as a marker for severe periodontal problems [45, 76]. One study did not use the Miller index score to assess tooth mobility, but mainly reported horizontal mobility less than 1 mm (score 1), between 1 and 2 mm (score 2) or more than 2 mm (score 3) [76]. The Miller index score was also combined with other parameters such as presence of calculus and bleeding on probing [45].
- Other measures were simply measuring pocket depth [55], assessing periodontal status by describing presence of calculus and bleeding on probing [45] or gingivitis assessment by measuring pocket depth, assessing bleeding, suppuration and/or tooth mobility class III [95].

### Plaque indices

Plaque indices used to describe oral health were used in 21 studies [5, 32, 39, 41, 50, 55, 58–60, 71, 72, 80–82, 86, 88, 93, 94, 99–101]. The (modified) plaque index, using grades 0–3, distinguished no plaque to visible layers of plaque [39, 41, 50, 60, 71, 80, 86, 88, 94, 99, 101]. A more detailed index was the Quigley-Hein plaque index, which includes all teeth except third molars [58, 86]. Each surface was scored between 0 (no plaque) and 5 (two-thirds of the surface). An index for the entire mouth was determined by dividing the total score by the number of surfaces examined. The mucosal-plaque score [93, 100] was a scoring system used for dentate and edentulous individuals. It registered changes in oral mucosa (i.e. normal presence, mild, moderate or severe inflammation) and plaque score, both on natural teeth and on removable dentures/fixed prosthodontics. The mucosal and plaque scores were combined to calculate the index.

The plaque control record used a plaque indicator on four sites of each tooth [32, 59, 81]. The plaque control record was calculated as the ratio of plaque-positive sites to all sites, expressed as a percentage. A different approach was only including plaque accumulation approximally, and reporting this as a percentage [100].

### Calculus indices

Calculus was scored in 5 studies.

Two studies used the Volpe-Manhold index, which quantifies calculus formation on the lingual surfaces of anterior lower teeth, recorded the calculus heights in millimeters [39, 86]. The calculus index scores calculus from 0 (no calculus) to 3 (supragingival calculus covering more than two-thirds of the cervical portion of the tooth) [43] or as present/absent [45]. Presence or absence of calculus was also recorded for each tooth and by dividing by the total number of teeth, resulting in a calculus score [94].

### Bleeding/gingival indices

In 7 studies bleeding indices were reported: the modified sulcus bleeding index [93], the papilla bleeding index [86], the gingiva bleeding index [32, 35, 98], the sulcus bleeding index [55], presence of bleeding after probing [45]. Gingival indices were used in 12 studies and included the gingival or gingivitis index [39, 50, 60, 62, 80, 86, 88, 99, 102] and the modified gingiva index [73, 82], to assess the visual appearance of inflammation of the gingiva (score 0–3 and score 0–4).

### Oral hygiene

Oral hygiene was assessed in 23 unique studies. The oral hygiene index (OHI) is designed for dentate persons and combines plaque and calculus indices [49, 86]. A shorter version is the simplified-OHI; 6 representative teeth were used instead of all sextants [40, 43, 54, 73, 83, 102], or the modified OHI, which used the summation of average debris index and calculus index [46]. Another instrument used was the Missisippi OHI, which used plaque disclosing agent, and divided each tooth in 5 sections, which were all scored [47]. In case of edentulous elderly a specific denture hygiene index could be used [32, 40, 41, 55, 58, 81, 83, 98], using either grades or a percentage to express cleanliness. A different method for determining oral hygiene was scoring food debris [86] or using the debris index [40, 43, 83, 90].

### Denture related parameters

Denture related parameters were used in 35 unique studies. Simply assessing the presence or absence of dentures was reported in 23 studies [27, 29, 38, 40, 42, 45, 48, 51, 60, 64, 65, 67, 70, 73, 77, 78, 81, 85–87, 90, 91, 103].

A slightly more detailed method was assessing the fit of the dentures, which was done in 10 studies [5, 51, 52, 56, 71, 84, 86, 94, 97, 104]. One study assessed the type, fit and condition of the denture by using the classification of Vigild [49], other studies evaluated the retention and stability, the quality of the denture of prosthetic need [72, 75, 79, 85].

Other objective oral health parameters used were in the domain of oral function, oral pathology and halitosis (Table 1). As they were not frequently used (usually reported in only 1 or a few studies) or were not standardized research parameters, they are not further described in this section.

2. Subjective parameters (Table 2).

Subjective parameters were used in 5 unique studies for dry mouth, 6 studies for oral health and 6 studies for oral pain. The xerostomia inventory [79] consisted of 11 items concerning dry mouth scored by the patient. The summation inventory consisted of 5 items related to dry mouth [40]. The xerostomic visual analog scale [39] focused one question: ‘how dry is your mouth?’; the patient’s answer was recorded as a continuous variable between 0 and 10. Reporting oral health problems was done by either questioning problems with chewing, swallowing and dry mouth [78] or problems with eating due to artificial teeth, chewing and xerostomia [108]. Other questionnaires focused on either dry mouth and oral motor function [105], oral function and oral problems [94], or only on oral symptoms, such as sensitive or broken teeth or bleeding gums [57] or oral pain, bleeding gums and tooth mobility [88]. Also reported are two validated questionnaires focusing on self-reporting oral discomfort (Oral Health Impact Profile: OHIP) and self-perceived oral health (Geriatric Oral Health Assessment Index: GOHAI) [61, 64, 65, 67, 72, 100, 101]. One study used a dental visit checklist to determine how often the dentist was visited in the past 2 years including the reason for dental visits [97].

Presence of previously experienced oral pain of discomfort required the input of the elderly participants and was used in 4 studies [46, 49, 84, 109]. In one study the orofacial-pain scale for the non-verbal individuals was used [40].

All studies used 2 or more parameters, subjective and/or objective, and usually of different domains. The same combination of parameters was never used.

3. Combined parameters (Table 3).

Combined parameters used a variety of assessments, objective as well as subjective, combined in one instrument to define oral health. In 8 studies the Oral Health Assessment Tool (OHAT) was used [27, 41, 103, 107, 110–113]. This validated tool focuses on 8 categories (lips, tongue, gums and tissues, saliva, natural teeth, dentures, oral cleanliness and oral pain) [114]. All categories were scored as healthy, changed or unhealthy. The Revised Oral Assessment Guide – Jonköping (ROAG(J)) is somewhat similar, as oral health is evaluated by assessing the condition of voice, lips, oral mucosa, tongue, gums teeth, saliva, swallowing, protheses/implants (grades 0–3) [25, 80, 81, 115, 116], however, this method is unvalidated. The OHAT and ROAG(J) are instruments developed for trained nursing staff, as is the Brief Oral Health Examination Status [122]. Other instruments were either modified or self-created instruments (asymptotic dental score [118], oral health examination instrument [109], clinical dental functionality score [96], oral tissue anomalies scale [94] or only used by nursing staff or research assistants (resident assessment instrument – minimum data set [96, 119, 120]).

### Oral health assessor

The assessor of oral health varied between the studies. Objective parameters were mostly assessed by dental professionals, but research examiners were used for assessment of salivary secretion and salivary flow rates [53, 105], oral malodour [107] and soft tissue lesions [104]. The dental professionals themselves were not calibrated, therefore there will always be an impact on the consistency of these parameters.

Subjective parameters required input of the patient, the patient or caregiver completed a questionnaire or scale. In some studies [46, 49, 109], the dental professional asked specific questions to the patient (for instance: were there recently dental pain complaints?) and recorded these answers. One study used the orofacial pain scale for the non-verbal individuals, which is specifically designed for examiners (or observers) [40]. The combined parameters were assessed by trained nursing staff as these parameters were designed to be used by non-dental care professionals. Of the combined parameters, the Asymptotic Dental Scale (ADS) [118] and Oral Health Examination scale [109] were assessed by dental professionals.

## Discussion

This scoping review on oral health assessment in institutionalized elderly showed that there is an enormous variability in parameters to define or describe oral health in this specific patient group. Among the objective parameters, there is great variability in interpretation of collective terms as oral (health) status, dental status, oral function and oral pathology and besides, a huge variability in methods to assess the same parameter. There is variability in the assessor too.

This study revealed 90 different parameters for determining oral health institutionalized elderly. Fifty of these parameters were solely used by one individual study. Only 4 parameters (4.4%) were frequently used, i.e. in 20 or more studies.The relevance of these frequently used objective parameters (DMFT/S, dental status (presence and number of teeth), plaque index and denture presence) in this specific patient group is discussed hereafter.

The DMFT/S provides information whether dental treatment has been done (presence of fillings) or if treatment is required (active decay). Indeed, active decay is an important aspect of oral health in this patient group, but it is questionable whether the amount of restorative treatment is relevant too, as elderly usually have a long treatment history. In addition, it can be argued if recording the absence or presence of teeth provides enough information on oral health as it does not provide any information on several other oral health aspects regarding pathology or functionality. The same accounts for denture presence; it does not inform on prosthesis quality and is not relevant for elderly with remaining teeth. Lastly, oral hygiene is an important aspect of oral health, but assessing the plaque index only is too little to qualify oral health in this patient group with often complex oral situations and an almost always inadequate level of oral hygiene.

Oral health described by periodontal parameters may give a better insight of oral health as periodontal disease is associated with inflammatory burden. The CPITN is most frequently used. This method is well suited as a screening tool to assess periodontal health. As it combines presence of periodontal pockets, gingival bleeding and calculus, it functions as a method to examine the periodontium. In combination with the plaque index, this instrument provides detailed information on periodontal health. However, in this complex patient group, periodontal screening with a periodontal probe cannot always be adequately performed as many elderly with complex care needs are uncooperative or in a difficult physical position for oral examination, such as patients in wheelchairs or lying in bed [5]. Other parameters to assess periodontal health, for example radiographic assessment, mobility of teeth, furcation involvement, gingival swelling, spontaneous bleeding or oral malodour may be more easily performed in this population.

Dental treatment need [27, 29, 46, 56, 84, 92], dental risk assessment [63], oral health status [87, 92, 97] and oral health index [71] are not frequently used but appear to be more valuable to assess oral health. Dental treatment need, however, only distinguishes need for treatment, which is a broad term and does not provide detailed information on oral health. Studies on oral health status use all their own methods, which often relies on the dentist’s judgement based on a few parameters, such as dental visits and presence of teeth [97] scoring oral care status of teeth and dentures [92]. Generally, these parameters are minimally described. Dental risk assessment [63] and oral health index [71] use a grading system to assess oral health based on a few parameters. The dental risk assessment does not inform on oral health status but mainly distinguishes older people ‘at risk’ and is therefore not suited to determine oral health. The oral health index [71] based on the presence of caries and root remnants, evaluation of periodontium, oral hygiene and denture seems better equipped to assess oral health. This method is, however, still in pilot study phase.

Interestingly, only two studies included radiographic assessment of oral health [29, 35], whereas in this patient group, radiographs can provide relatively simple an objective overview of multiple oral health problems: presence of caries, periodontal problems as subgingival calculus and furcation involvement, periapical granulomas, quality of previously performed endodontic treatments, oral pathology and the presence of root remnants or impacted teeth.

Subjective parameters evaluate oral health using self-reported input of the elderly. Frequently reported subjective parameters are oral dryness, oral symptoms such as sensitive teeth, oral pain or oral health-related quality of life. These parameters provide important additional information for the dental professional; by assessing someone’s subjective oral health complaints, specific objective parameters can be used to evaluate and qualify their oral health.

Validated questionnaires on self-reported oral health are the OHIP-49 and GOHAI [100], focusing on several oral health-related items and their impact on the elderly’s wellbeing (quality of life). As the goal of these questionnaires is assessing quality of life, these instruments are not well suited to score oral health objectively [125].

The category of combined instruments comprises the validated OHAT [27, 41, 103, 107, 110–113], created for nursing staff scoring oral health items by appearing ‘healthy’ or ‘unhealthy’. Although the OHAT, together with a newer version of the oral health-related section of the RAI-MDS (the ohr-InterRAI) were considered to have sufficient content validity [126], all oral health assessment instruments for non-dental professionals showed narrow content, poorly defined constructs for measurement, and psychometric weaknesses [127, 128]. Indeed, there are reported differences between the assessment of oral health of institutionalized elderly by dental professionals versus non-dental professionals [129]. Therefore, it is preferred that oral health assessment in this patient group is performed by dental professionals.

Limitations associated with this study are the wide range of different aspects of oral health in institutionalized elderly, and the huge variability among the parameters described for all these different aspects. Data synthesis and -presentation are therefore challenging, and the overview of oral health parameters is comprehensive. The lack of calibration of dental professionals in the included studies makes it impossible to value the different parameters and to formulate clear recommendations.

### Concluding remarks

It is concluded that in institutionalized elderly, the huge variability in methods to determine oral health, makes it impossible to compare studies on oral health and the effect of (preventive) interventions in this vulnerable patient group. Given the concerns about the effect of poor oral health on quality of life and healthy ageing in a physical and mental context and the newly formulated goals of global institutions as The World Health Organization and The United Nations Decade of Healthy Ageing (2021–2030) [20], this is problematic.

There is an urgent need for an adequate and uniform parameter for oral health determination in institutionalized elderly, to aid the planning and commissioning of future research and patient care.

Oral health assessment in institutionalized elderly should ideally be easily performed, objective, assessed by a dental professional, and reflect on items that may interfere with quality of life or general health such as pain, inflammation, oral pathology and oral function.

### Supplementary Information

**Supplementary file 1.**

**Supplementary file 2.**

**Supplementary file 3.**

## Data Availability

The datasets used and/or analyzed during the current study available from the corresponding author on reasonable request.

## Abbreviations

DMFT/S: Decayed, Missed, Filled Teeth/Surfaces
CPITN: Community Periodontal Index of Treatment Needs
OHI: Oral Hygiene Index
OHIP: Oral Health Impact Profile
GOHAI: Geriatric Oral Health Assessment Index
OHAT: Oral Health Assessment Tool
ROAG-J: Revised Oral Assessment Guide Jonköping
RAI MDS: Resident Assessment Instrument/Minimum Data Set
