# Supplementary material for: Oral health assessment in institutionalized elderly: a scoping review
Source: BMC Oral Health. 2024 Feb 24;24:272. doi: 10.1186/s12903-024-04025-y (PMC10893687; doi:10.1186/s12903-024-04025-y)
Supplement: Supplementary file 1 [file 12903_2024_4025_MOESM1_ESM.docx]

**Supplementary file 1: Search strategy.**

| Online library | Search terms | Hits |
| --- | --- | --- |
| Medline | ((oral health [MeSH Terms])) AND ((elderly [MeSH Terms]) OR (Aged [MeSH Terms])) AND ((nursing home [MeSH Terms] OR (institutionalized [tiab])) | 343 |
| Cinahl | (oral health) and (elderly) and ((nursing home) OR (institutionalized)) | 5 |
| Cochrane Library | (([Oral Health] MeSH) OR (oral health):ti,ab,kw) AND (([Aged] MeSH) OR (elderly):ti,ab,kw) AND (([Nursing home] MeSH) OR (nursing home):ti,ab,kw OR (institutionalized:ti,ab,kw)) | 149 |
