# Supplementary material for: Oral health assessment in institutionalized elderly: a scoping review
Source: BMC Oral Health. 2024 Feb 24;24:272. doi: 10.1186/s12903-024-04025-y (PMC10893687; doi:10.1186/s12903-024-04025-y)
Supplement: Supplementary file 2 [file 12903_2024_4025_MOESM2_ESM.docx]

**Supplementary file 2:**
