# Supplementary material for: Oral health assessment in institutionalized elderly: a scoping review
Source: BMC Oral Health. 2024 Feb 24;24:272. doi: 10.1186/s12903-024-04025-y (PMC10893687; doi:10.1186/s12903-024-04025-y)
Supplement: Supplementary file 3 [file 12903_2024_4025_MOESM3_ESM.docx]

**Supplementary file 3: Continents and number of high-, middle-, and low-income countries, based on New World Bank country classification (2022-2023) (126)**

| Continent | Number of studies in low-income countries | Number of studies in middle-income countries | Number of studies in high-income countries |
| --- | --- | --- | --- |
| Asia | 0 | 4 | 8 |
| Europe | 0 | 1 | 48 |
| Northern-America | 0 | 0 | 20 |
| Southern-America | 0 | 4 | 1 |
